# High-resolution Imaging of the Human Cochlea through the Round Window by means of Optical Coherence Tomography

**DOI:** 10.1038/s41598-019-50727-7

**Published:** 2019-10-03

**Authors:** Anastasiya Starovoyt, Tristan Putzeys, Jan Wouters, Nicolas Verhaert

**Affiliations:** 1 Research Group Experimental Oto-Rhino-Laryngology, Department of Neurosciences, University of Leuven, Leuven, Belgium; 2 Department of Otorhinolaryngology, Head and Neck Surgery, University Hospitals of Leuven, Leuven, Belgium; 3 Laboratory for Soft Matter and Biophysics, Department of Physics and Astronomy, University of Leuven, Leuven, Belgium

**Keywords:** Translational research, Imaging and sensing

## Abstract

The human cochlea is deeply embedded in the temporal bone and surrounded by a thick otic capsule, rendering its internal structure inaccessible for direct visualization. Clinical imaging techniques fall short of their resolution for imaging of the intracochlear structures with sufficient detail. As a result, there is a lack of knowledge concerning best practice for intracochlear therapy placement, such as cochlear implantation. In the past decades, optical coherence tomography (OCT) has proven valuable for non-invasive, high-resolution, cross-sectional imaging of tissue microstructure in various fields of medicine, including ophthalmology, cardiology and dermatology. There is an upcoming interest for OCT imaging of the cochlea, which so far was mostly carried out in small animals. In this temporal bone study, we focused on high-resolution imaging of the human cochlea. The cochlea was approached through mastoidectomy and posterior tympanotomy, both standard surgical procedures. A commercially available spectral-domain OCT imaging system was used to obtain high-resolution images of the cochlear hook region through the intact round window membrane in four cadaveric human temporal bones. We discuss the qualitative and quantitative characteristics of intracochlear structures on OCT images and their importance for cochlear implant surgery.

## Introduction

Disabling hearing loss affects over 6% of the world population, yet effective therapeutic options are limited (World Health Organization)^1^. Malfunction of the cochlea is especially challenging to treat, since in this case the hearing aids cannot sufficiently restore the auditory function. Over the past four decades, the development of the cochlear implant (CI) enabled hearing restoration in more than 300,000 patients with hearing loss due to cochlear pathology^2,3^. Recently, the indications for CI have also been extended to subjects with residual hearing. In comparison to deaf CI recipients, these patients achieve better speech recognition and greater appreciation of music^3^.

The CI consists of an external and an internal part. The former, contains a speech processor, which converts recorded sound into an electrical signal. The latter, decodes this signal and sends electrical pulses to an electrode array, which is located inside the cochlea. The electrical pulses directly stimulate the auditory nerve, bypassing the malfunctioning cochlea^3^.

**Figure 1:**
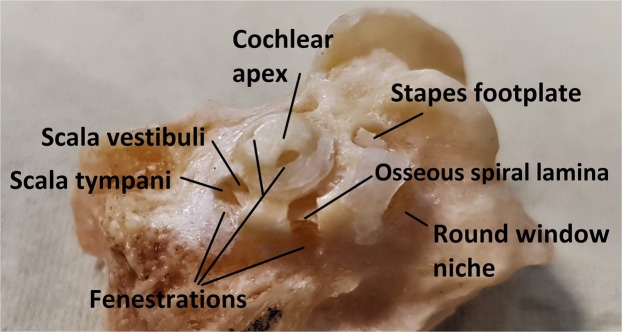
Fenestrated cochlea of the TB4, left fresh-frozen human temporal bone. The important anatomical structures are indicated. Scala vestibuli and scala tympani are separated by the osseous spiral lamina.

The implantation of the internal part requires surgery. Hereby, an opening is drilled in the patient’s skull to insert the electrode into the scala tympani (ST) compartment of the cochlea (Fig. 1). There are in total three fluid-filled compartments inside the cochlea: ST, scala vestibuli (SV) and scala media (SM). The SM is the smallest compartment and contains the auditory sensory epithelium. The division of the cochlear compartments and their respective fluids is crucial for the hearing function. The ST is separated from the other compartments by a thin bony plate – the osseous spiral lamina (OSL) – and soft tissue structures – the basilar membrane (BM) and the spiral ligament (SL). The auditory nerve fibers pass through the OSL towards the auditory nerve. The fluid-filled cochlea is surrounded by dense cortical bone, except for two small windows (Fig. 1). The oval window is connected to the SV and it is covered by the thin bony footplate of the stapes, one of the three middle ear ossicles. The round window, which is the entrance to the ST, contains a 70 µm thick membrane, called the round window membrane (RWM)^4–7^. These two openings are the only visible parts of the cochlea during CI surgery.

**Figure 2:**
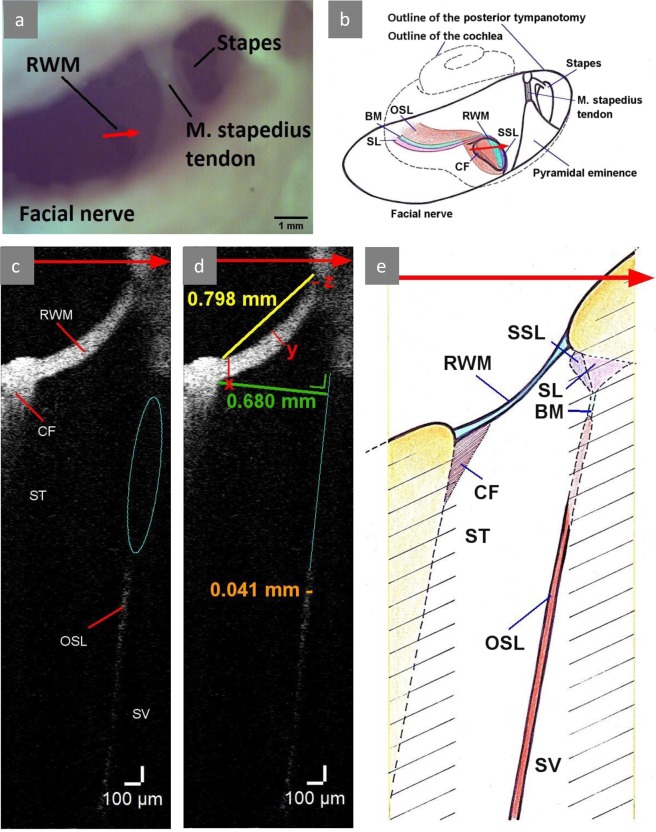
OCT imaging in the TB1, left fresh-frozen human temporal bone. (**a**) Photograph of the microscopic image of posterior tympanotomy. Red arrow shows the position of the cross-section plane on the RWM, the arrow point corresponds to the superior margin of the RWM and the right side of the cross-sectional OCT image. (**b**) Schematic drawing of the posterior tympanotomy in (**a**). The structures, visible on the microscopic image in (**a**) are drawn with a continuous line. The rough contour of the membranous cochlea is drawn with a dashed line. The three-dimensional course of the intracochlear structures is depicted in different colors: (orange) OSL, (green) BM, (pink) SL, (purple) SSL. The position of the CF is drawn in brown at the anteroinferior edge of the RWM. Note the changing orientation of the OSL, the BM and the SL. (**c**) Two-dimensional OCT scan through the RWM. The proximal part of the OSL, the BM, the SL and the SSL could not be visualized due to the shadow of the bone of cochlear capsule (blue ellipse). (**d**) Measurements of the RWM width (yellow), RWM thickness (red), ST width (green) and OSL thickness (orange) in this section were taken in ThorImage OCT 4.4 software with an assumed refractive index of 1.00. The RWM thickness was measured in three points: x = 0.118 mm, y = 0.106 mm, z = 0.060 mm. We measured the ST width as the largest distance between the proximal cochlear lateral wall and the extension of the osseous spiral lamina (thin blue line), perpendicular to the osseous spiral lamina. After correction for the refractive index of the intracochlear fluid, 1.33^45^, this results in an actual ST width of 677 mm. (**e**) Schematic drawing of the cross-section, corresponding to the OCT image in (**c**). The structures, visible on the OCT image in (**c**) are drawn with a continuous line. The structures of the corresponding cross-section, which are not visible on the OCT image are drawn with a dashed line. The yellow lines represent the borders of the scanned area. The intracochlear structures have been depicted in the colors, corresponding to (**b**). The hatched area represents the shadow of the cochlear capsule, which hides the structures underneath it. BM: basilar membrane, CF: crista fenestra, OSL: osseous spiral lamina, RWM: round window membrane, ST: scala tympani, SV: scala vestibuli.

There are two approaches for electrode insertion in CI surgery: the round window approach, whereby the insertion is performed through the RWM; and the cochleostomy approach, whereby the electrode is inserted through into the ST through a surgically drilled opening. The electrode passage through the proximal part of the cochlea, known as the ‘hook region’, is crucial for the rest of the insertion. The hook region is characterized by its fish-hook shape and its distinctive anatomy, compared to the rest of the cochlea: all cochlear compartments rotate 90° in the proximal hook region, whereby the orientation of the OSL, the BM and the SL changes from horizontal to vertical^8,9^ (Fig. 2b). Despite this rotation, the ST follows an approximately straight line until the first cochlear turn, while it gains an increasing curvature further in the cochlea^8^. The proximal ST also contains two bony protrusions, which are only present in the hook region: the secondary spiral lamina (SSL), which is located on top of the SL at the superior rim of the RWM^9,10^ and the crista fenestra, which is a bony crest at the anteroinferior rim of the RWM (Fig. 2b,e). The CF protrudes into the ST and can cause a variable narrowing of its proximal diameter. This narrowing can lead to the deviation of the electrode array during the insertion and trauma to the intracochlear structures^11,12^.

Insertion trauma can impair the hearing outcome^13^ through destruction of the barriers between the cochlear compartments, damage to the auditory neurons and reactive inflammation and fibrosis^3,12,14^. In patients with residual hearing, this leads to an even more adverse outcome^15,16^. A large portion of insertion trauma occurs within the first 180° of the cochlea^8,17,18^. Atraumatic insertion requires electrode passage in line with the ST trajectory^8,19,20^. The cochleostomy enables a more controlled proximal insertion trajectory^19^, but recent studies show that drilling of an entrance to the ST inevitably causes trauma to the intracochlear microstructures^21,22^. In view of the growing importance of atraumatic CI surgery, it is now recommended to insert the electrode through the RWM, when possible^9,23^.

The main cause of insertion trauma is the lack of visual guidance for the electrode insertion, since the surgical view enables only the visualization of the entrance to the ST (the cochleostomy or the RWM). Additionally, current clinical imaging techniques do not provide sufficient spatial resolution for reliable evaluation of the individual ST compartment and the CF region^12,24^. While implementation of soft surgery techniques and modified electrode design have partially reduced the incidence of insertion trauma^3,17,20,25^, intracochlear trauma still occurs in up to 40% of the electrode insertions^15,26^. Thus, there is a need for a high-resolution imaging technique, which can be safely applied in otological clinical practice.

Optical coherence tomography (OCT) is a non-invasive, optical imaging modality, which enables cross-sectional and three-dimensional tomographic imaging of tissue microstructure with a resolution of 1 to 15 µm^27^. The images are reconstructed based on the magnitude and the time of flight of backscattered near-infrared, low-coherence light by means of interferometry^28,29^. OCT can safely be applied *in vivo* and has already become the standard of care in clinical ophthalmology and dermatology. This imaging technology also promises to have a powerful impact in many other medical fields such as otology^27^. So far, OCT imaging of the cochlea has been mostly carried out in rodents. Their cochlear capsule is sufficiently thin to be penetrated by the near-infrared light waves, which enables imaging of the complete cochlea with OCT^24,30–36^. Important intracochlear structures have been visualized: the different scalae, the BM, the SL, the OSL, the sensory epithelium and the SSL^24,30–36^.

In humans, OCT has already successfully been applied for *in vivo* middle ear imaging through the tympanic membrane^37,38^, while only a few studies report (*in vitro*) OCT imaging of the human cochlea^32,39–42^. One of the main challenges for intracochlear OCT imaging of the human cochlea is the thick cochlear bone. Some authors have removed or thinned parts of the bone^40–42^, while others proposed imaging with a thin OCT probe, inserted inside the cochlea^32^. There is a great interest for the use of OCT in CI surgery^32,41^ and studies have shown that this imaging technique can provide guidance on the drilling of the cochleostomies^40,43^.

The purpose of this study is to evaluate the capability of OCT to provide guidance for the electrode insertion in the round window approach. In this approach, it is important to assess the CF region and the trajectory of the ST in order to avoid trauma during the insertion of the electrode array^8,12,19^. We used a commercially available OCT imaging system from Thorlabs with a stationary scanner. OCT imaging was performed through the RWM, the natural access for electrode insertion. This bypassed the need to remove the cochlear bone or to insert an OCT probe into the cochlea. In the rest of this paper, we use the term ‘transmembrane imaging’ for imaging through the RWM.

We argue that OCT imaging can guide the electrode insertion through the RWM, if the following three hypotheses prove to be true:

- Hypothesis 1: Transmembrane OCT imaging can resolve anatomical structures of the hook region in a human cochlea.
- Hypothesis 2: The visualized anatomical structures of the hook region are relevant for CI surgery.
- Hypothesis 3: Transmembrane OCT imaging enables evaluation of the insertion trajectory in a surgically accessed cochlea.

In the following sections, we report our first results on transmembrane intracochlear OCT imaging in four human temporal bones (TBs). Subsequently, we discuss the implications of our research for CI surgery.

## Results

**Figure 3:**
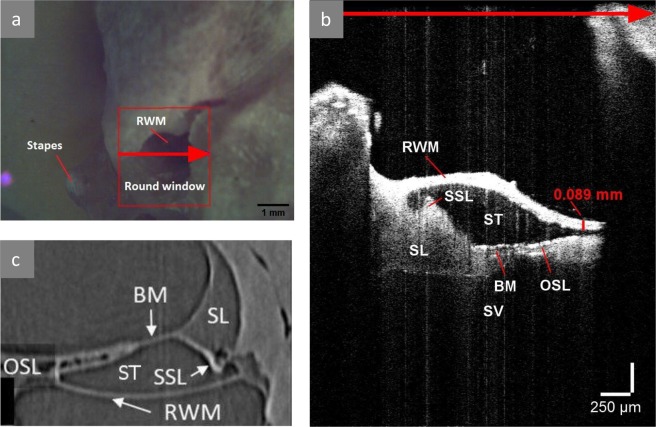
OCT imaging in the TB3, right fixed human temporal bone. (**a**) Screen shot of the RWM with the projected plane of cross-sectional imaging. Red rectangle shows the area of the three-dimensional OCT scan. Red arrow shows the position of the cross-section plane (**b**) on the RWM, the arrow point corresponds to the inferior margin of the RWM and right side of the cross-sectional OCT image. (**b**) OCT image of the perpendicular section through the RWM shows the proximal part of the ST, SV and the micro-anatomical structures in between. The thickness of the RWM (red value) at the inferior margin was measured in ThorImage OCT 4.4 software with an assumed refractive index of 1.00. This position was chosen for thickness measurement, because it is located in the central part of the RWM and the thickness in this point consists exclusively of the axial component. (**c**) Cross-sectional synchrotron radiation phase contrast image through RWM of a right fixed human temporal bone as published by Agrawal *et al*. in 2018. (Adapted with permission from Open Access ref.^10^) is similar to (**b**), when rotated over 180°. BM: basilar membrane, OSL: osseous spiral lamina, RWM: round window membrane, SL: spiral ligament, SSL: secondary spiral lamina, ST: scala tympani, SV: scala vestibuli.

We produced two- and three-dimensional transmembrane OCT images in four human temporal bones. The central cross-sections of the three-dimensional OCT images are summarized in Supplementary Fig. 1. Imaging of the fresh-frozen TB1 (Fig. 2) and the fixed TB3 (Fig. 3) resulted in the most characteristic and detailed images, as will be described in this section.

### OCT imaging in an isolated cochlea

In TB3 and TB4, the accessibility of the cochlea was maximized in order to study the resolution potential of the OCT system TEL220C1 for transmembrane intracochlear OCT imaging (hypothesis 1 and 2). Images were recorded normal to the plane of the RWM. Figure 3a shows the microscopic view, corresponding to cross-sectional OCT imaging. The incidence angle, which was used in this microscopic view, was selected for the optimal visualization of the different intracochlear structures (Fig. 3b): the SSL, the SL, the BM and the OSL. The BM spans between the SL and the SSL; all these structures are parallel to the global plane of the RWM. The SL is attached to the lateral cochlear wall and forms the SSL at the superior rim of the RWM. On OCT images, the SSL can be distinguished from the SL by its higher contrast, due to the denser, bony structure. The OSL is the thinnest at the border with the BM, where its two-layer structure can be clearly visualized; further from the BM, the lowest bony layer of the OSL can no longer be distinguished due to scattering and attenuation.

In TB4, we observed that a fluid-filled cavity was formed between the layers of the RWM (Supplementary Fig. 1, TB4, cross-section x and cross-section y). Most likely, this was due to degradation of the loose connective tissue inside the RWM, caused by dehydration and subsequent moisturizing of the un-fixed TB4.

### OCT imaging in a surgically accessed cochlea

**Figure 4:**
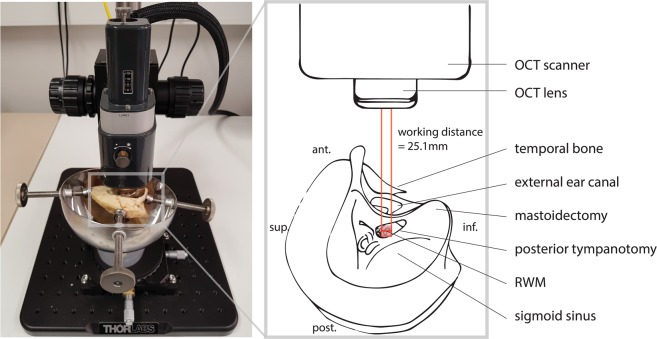
Setup for transmembrane OCT imaging in a surgically accessed cochlea. The photograph on the left illustrates the setup in the right-sided TB2. Next to it, we provide a schematic overview of the main elements: the OCT scanner and lens, the optical path and the temporal bone (with its orientation). The access to the RWM is gained through surgically drilled openings in the skull: mastoidectomy and posterior tympanotomy. The working distance between the OCT lens and the RWM, which is in focus, is 25.1 mm. The optical path and the cross-section in the center of the RWM are displayed in red. The rectangular field of view of the three-dimensional OCT scan (pink) contains the full RWM. ant. = anterior, inf. = inferior, post. = posterior, RWM: round window membrane, sup. = superior.

In TB1 and TB2, we focused on the possibilities of the setup in a surgically accessed cochlea (hypothesis 1 and 2) and on its utility for CI surgery (hypothesis 3). The access to the cochlea was gained through drilling of a mastoidectomy and a posterior tympanotomy in the temporal bones, both standard surgical procedures in cochlear implantation (Fig. 4). Figure 2a shows the microscopic view, produced by cross-sectional OCT imaging of TB1. The incidence angle was chosen in such way that the ST and the OSL could maximally be visualized through the RWM. This optical path (Fig. 4) enabled transmembrane imaging of the proximal hook region with the ST, the OSL, the SV and the CF region (Fig. 2c). It is worth noticing that the inferior part of the RWM has a higher reflectivity, than the superior part. This is caused by its perpendicular orientation towards the light beam and the detector, in contrast to the superior part, which is almost parallel to the light beam. The most proximal parts of the OSL, the BM, the SL and the SSL could not be visualized in the surgically accessed cochleae, as these structures were located under the cochlear bone at the superior edge of the RWM (Fig. 2b,c,e). More concretely, the cochlear bone scatters the near-infrared light and limits its penetration depth, hiding the underlying structures under its ‘shadow’ (Fig. 2d,e).

We observed that even though the surgical access to the RWM in TB2 was wider than in TB1, the access for transmembrane imaging was substantially worse in TB2. As a consequence, the posterior border of the RWM with the underlying ST could not be visualized (Supplementary Fig. 1, TB2, cross-section y). This was caused by an anteriorly displaced large brain vein, called sigmoid sinus^44^, which narrowed the surgical access and thereby prohibited optimal positioning of the objective lens inside the skull opening (Fig. 4).

In order to assess the insertion trajectory (hypothesis 3), we evaluated the visibility of the CF region (Fig. 2c). Optimal visualization of the CF was achieved with the incidence angle, which was parallel to the OSL. We determined the width of the ST, the diameter of the RWM and the thickness of the OSL (Fig. 2d) in the cross-section with the maximal superior-inferior ST diameter. As previously mentioned, OCT images are reconstructed based on the magnitude and the time of flight of backscattered light^28,29^. Therefore, the axial component on raw images represents the optical path length (OPL) and not the actual geometric distance, unlike the lateral component. The OPL is defined by the transit time of light as *OPL* = *n* × *d*, where *d* is the geometric distance and *n* is the refractive index of the medium^27^. When the refractive index of a structure is larger, it results in a higher transit time of light through this structure and therefore a larger OPL. On OCT images, this leads to increased axial dimensions of the structures, in proportion to their respective refractive index. This explains the gradual decrease of the RWM thickness between the inferior and the superior margin in Fig. 2c,d (decrease of the axial component). The ST width was obtained by correcting the axial component of the measurement for the refractive index of the intracochlear fluid, 1.33^45^. The RWM width was measured in air medium; in air, *n* = 1 and therefore *OPL* = *d*. The OSL thickness was measured exclusively in the lateral direction and thus did not require further correction.

### Estimation of the refractive index of the RWM

Accurate assessment of the optimal insertion vector requires correct representation of the intracochlear anatomy and thus correction of the refraction artifacts. However, the refractive indices of the human cochlear structures are not known, except for the refractive index of the intracochlear fluid. We assessed the refractive index of the RWM, based on the measured RWM thickness in the axial direction (*OPL*) and the reported value for the RWM thickness, which is 67.4+/−14.3^5^ (*d*): *n* = *OPL/d*. In the fresh-frozen TB1, the OPL was 118 (Fig. 2d, point x) and the calculated range for the refractive index of the RWM was [1.4; 2.2]. In the fixed TB3 (Fig. 3b), the calculated range was [1.1, 1.7].

## Discussion

The purpose of our study was to investigate if OCT imaging can be used as a guidance tool for the electrode insertion through the RWM. We formulated three hypotheses in order to assess the benefit of OCT imaging for CI surgery. We tested these hypotheses by performing transmembrane OCT imaging on four human temporal bones.

Our results showed that transmembrane OCT imaging is able to resolve intracochlear anatomy of the proximal hook region. The large intracochlear compartments (SV and ST) and the OSL could be imaged in all specimen (Figs 2–3, Supplementary Fig. 1). In the isolated cochleae of TB3 and TB4 (Fig. 3, Supplementary Fig. 1), OCT imaging was performed normal to the plane of the RWM. This enabled detailed visualization of the ST, the SV and the separating structures between these compartments: the soft BM and SL, as well as the bony SSL and the two-layers of the OSL. To the best of our knowledge, these are the first OCT images of the human SSL. They were comparable with synchrotron radiation phase contrast images, recently published by Agrawal *et al*.^10^ (Fig. 3c), and with the OCT images of the gerbil SSL, published by Cooper *et al*.^46^. However, synchrotron radiation phase contrast imaging of the human cochlea is only possible in small samples of temporal bones and not *in vivo*^10,47^. Therefore, OCT is currently the only safe, non-invasive technique that enables visualization of the SSL and can be applied in clinical practice. In the surgically accessed cochleae of TB1 and TB2 (Fig. 2, Supplementary Fig. 1), the intracochlear anatomy was imaged under a different angle. This optical path enabled the evaluation of the CF region and the proximal ST trajectory, while the BM, the SL and the SSL remained hidden behind the cochlear wall. In all temporal bones, we observed that the visualized structures and their aspect depend on the incidence angle, the optical properties of the tissues and the structures above it and the imaging plane for interference. Putting the tissue of interest parallel with the light beam and bringing it in optical focus improves the signal-to-noise ratio and enhances the resolution. The incidence angle for transmembrane OCT imaging should be chosen depending on the purpose of the study. For intracochlear diagnostics, morphological and functional study of the cochlea, the emphasis lies on the detailed visualization of the anatomical structures. In this case, the optimal incidence angle is normal to the plane of the RWM. In a surgically accessed cochlea, this angle could be achieved with an OCT-catheter as described by Lin *et al*.^32^ or an angled endoscope as proposed by Fujita *et al*.^48^. For guided electrode insertion, it is more important to visualize the ST trajectory, along with its possible obstacles, than the individual intracochlear structures. In this case, the optimal incidence angle can easily be achieved with a stationary OCT setup, also in a surgically accessed cochlea.

The visualized anatomical structures were relevant for CI surgery. Whenever there is electrode insertion trauma, the BM, the OSL or the SL are damaged^8,22,49^. In prominent trauma, there can even be electrode excursion from the ST into the SV compartment. This electrode displacement leads to complete loss of residual hearing^50^. The importance of the SSL anatomy in atraumatic and hearing preserving CI surgery has recently been discussed by Agrawal *et al*.^10^. This structure provides additional support for the BM, increasing its stiffness and supporting the hearing function in animals with high-frequency hearing, such as bats^10,51,52^. In humans, the SSL is relatively underdeveloped, but it may similarly suspend the BM or contribute to the filtering of high-frequency sounds in the hook region^10,11^. The CF anatomy varies among individuals and it is very important for the electrode insertion through the RWM. Approximately 10% of people have a prominent CF^6^, which can cause direct deviation of the inserted electrode and trauma to the modiolus, which is the central axis of the cochlea, and the OSL; both of them contain the auditory nerve fibers^11,12,53,54^. In patients with a prominent CF, it may be safer to insert the electrode array through a cochleostomy rather than the RWM^12^. So far, no clinical imaging technique was able to visualize the CF^12^. We were able to show that OCT can resolve this unpredictable and highly important region for CI surgery (Fig. 2c) and that the proximal ST diameter can be determined on the acquired OCT images (Fig. 2d). In our samples, the CF did not cause substantial narrowing of the ST, which can otherwise hinder the electrode insertion through the RWM. Further studies with a larger sample size are certainly needed to further investigate the variability of the CF region and its influence on the preferred surgical approach.

Finally, we demonstrated that transmembrane OCT imaging with a stationary scanner enables evaluation of the insertion trajectory, in the surgically accessed cochleae. We visualized the ST, the SV and the OSL between these compartments in TB1 and TB2. Here, the stationary scanner was positioned in such way that the incidence angle was approximately in line with the ST. This angle enabled evaluation of the ST trajectory, based on the position and the orientation of the OSL. We hypothesize that the insertion vector should be chosen parallel to the visible OSL. Firstly, this will prevent trauma to the proximal OSL itself and to the structures, which are adjacent to it: the BM, the SL and the SSL (Fig. 2e). Furthermore, since the proximal ST follows an approximately straight line^8^, the choice of the correct insertion vector will guarantee atraumatic passage of the electrode until the first cochlear turn. Finally, studies suggest that optimization of the insertion vector can also prevent electrode dislocation near 180°^17,18^. In future research, also the optimal distance between the visualized OSL and the electrode should be determined. Studies have reported that the electrode should be inserted through the center of the ST^8,19,20^.

Based on our findings, we conclude that transmembrane OCT imaging is a very promising tool for CI surgery. Intra-operative visualization of the CF region would enable the surgeon to make better-informed decisions on the approach for the electrode insertion (cochleostomy or round window) in an individual patient. Furthermore, it facilitates assessment of the optimal insertion vector, prior to the actual insertion of the electrode. This can lead to significant decrease in insertion trauma, improved hearing outcome and increased rates of preserved residual hearing in CI recipients. Should a cochleostomy be needed, this same technology can be used to guide the drilling and minimize the trauma^40,43^. In addition, the incidence angle for OCT imaging approximates the viewing angle with a surgical microscope, which simplifies the anatomical orientation for the surgeon and allows for transmembrane imaging with a stationary OCT microscope.

Just like other imaging techniques, OCT is not immune to artifacts. Bone tissue causes scattering and attenuation of the near-infrared light, limiting its penetration depth and inducing a ‘shadow’ artifact on the underlying structures^27^. Therefore, interpretation of the intracochlear images requires profound knowledge of the intracochlear anatomy, as well as the anatomical variants. OCT images also contain refraction artifacts. As discussed in the results section, refraction leads to increased axial dimensions of the structures, proportional to their respective refractive index^27^. Furthermore, light waves through a not horizontal surface change direction according to Snell’s law^27^. This can lead to distorted representation of the position and the orientation of the OSL on OCT images and thus an incorrect assessment of the optimal insertion vector. Correction of refraction artifacts is non-trivial and usually requires knowledge of the refractive indices of the imaged tissue^55^. In this study, we provided the first rough estimation for the refractive index of the RWM: in range of [1.4; 2.2] for fresh-frozen and in range of [1.1, 1.7] for fixed temporal bones. Further studies are required to accurately determine the refractive indices of the intracochlear structures and to investigate the impact of the refraction artifacts on the predicted insertion vector.

The experiments in this study were conducted, using a commercially available spatial domain OCT system from Thorlabs with a stationary scanner and wavelength of 1300 nm. This setup combined high spatial resolution with sufficient depth penetration at a high image acquisition speed, making it suitable for evaluation of the electrode insertion trajectory. The acquired images demonstrate that transmembrane imaging in a surgically accessed cochlea can be performed with a stationary OCT scanner. The main limitation of the current setup for intraoperative application is the short working distance of the objective lens at 25.1 mm, since the optimal placement of the lens depends on the patient’s skull anatomy at this distance from the RWM (Fig. 4). This limitation can be overcome by using an objective lens with a larger working distance. In surgical settings, a stationary OCT set-up can be incorporated into an OCT-adapted microscope^40^.

## Conclusion

The purpose of this study was to evaluate the feasability of OCT as a guidance tool for electrode insertion in order to reduce insertion trauma in cochlear implant surgery. To this end, OCT imaging was performed in four human temporal bones, through an intact round window membrane. The produced images demonstrate that OCT enables visualization of (a) the intracochlear anatomy in the proximal ‘hook region’ and (b) the trajectory of the scala tympani. Intra-operative use of this imaging technique can lead to better-informed decisions when selecting the approach for electrode insertion (cochleostomy or round window), along with the optimal insertion vector, thereby decreasing the electrode insertion trauma. A natural progression of this study is to determine the refractive indices of the intracochlear structures and to investigate the impact of the refraction artifacts on the predicted insertion vector.

## Methods

### Temporal bone extraction and preservation

Four human cadaveric temporal bones (TBs) were obtained from the Vesalius Institute of the University of Leuven. All TBs were harvested post mortem from individuals who voluntarily donated their body to research in their testament. The donors were anonymous and no biographical donor data were known. Harvesting and use of the TBs was conducted in accordance with the Helsinki declaration and approved by the Medical Ethics Committee of the University Hospitals of Leuven (approval No. NH019 2016-06-04). We used two fixed (TB 2, TB 3) and two fresh-frozen (TB 1, TB 4) TBs. 72 hours elapsed between patient death and fixation or freezing respectively. During this time period, the fresh human cadaver was preserved at 2 °C. Fresh-frozen TBs were extracted with a hole saw with inner diameter of 7.5 mm from fresh human cadavers and subsequently preserved in a small plastic bag at −15 °C. For the experiments, frozen TBs were thawed at 2 °C during 48 hours. Fixed TBs were extracted from human cadavers, which were embalmed with a phenol – ethanol – glycerin – chloral hydrate solution within 72 hours after death, in accordance to our institutional guidelines. Extracted fixed TBs were preserved in a small plastic bag at 2 °C. No additional processing, such as staining, sectioning or decalcification, was performed on the TBs.

### Temporal bone dissection

TBs were dissected to a various degree. The number of the TB corresponds to the degree of dissection. An overview of the TBs and their dissection is provided in Supplementary Table 1. TB1 was dissected in accordance to the standard procedure for CI surgery^56^ (mastoidectomy, posterior tympanotomy, removal of the bony overhang surrounding the RWM). In TB2, the tympanic segment of the facial nerve was removed in order to obtain an enlarged posterior tympanotomy. This was a step in between, before complete simulation of the surgically accessed cochlea in TB1. Maximal access to the cochlea was achieved through isolation of the inner ear in TB3 and TB4. In these TBs, the external ear canal was drilled away and the tympanic membrane was carefully removed, together with the malleus and the incus; the stapes was preserved. Then, the skeletonized cortical bone overlying dura mater and sigmoid sinus was also drilled away. Finally, the otic capsule of the inner ear was thinned with 3.0 mm, 2.0 mm and 1.0 mm diamond burrs, until the contours of the membranous labyrinth (the semicircular canals and the cochlea) became visible through the cortical bone. In TB4, fenestrations were made in the thinned cochlear capsule (Fig. 1) in order to enable direct comparison of the acquired cross-sectional OCT images with the exposed intracochlear structures, evaluated under Zeiss operating microscope. This bypassed the need for histological preparation during the experiments. The fenestrations were drilled with 2.0 mm and 1.0 mm diamond burrs. They were approximately 2 mm wide and opened both the ST and the SV. We made three fenestrations in the basal turn and two fenestrations in the second turn of the cochlea. The distance between the fenestrations was approximately 3 mm. These positions were chosen in such way that the ST and the SV could be visualized over their full length, thereby improving three-dimensional orientation in an isolated inner ear. Due to the presence of fenestrations in the cochlea of TB4, the intracochlear structures dried out prior to OCT imaging. In order to improve the resolution, TB4 was moisturized with tap water before imaging.

### OCT imaging

TBs were scanned with an SD OCT imaging system (Telesto TEL220C1; Thorlabs, Lübeck, Germany)^57^ with the following characteristics: central wavelength of 1300 nm and axial resolution of 5.5 μm in air. We used objective lens LSM03 from Thorlabs with magnification of 5.0x, working distance of 25.1 mm and lateral resolution of 13 μm. The system contained a stationary scanner, adjustable scanning stand and translational stage. The stationary setup approached the operating microscope view during the CI surgery. The refractive index was set at 1.00 (refractive index of air) for simplification of the refractive index computation on OCT images. Furthermore, this allowed for correct measurement of the external RWM diameter (Fig. 3d), because the OCT imaging was performed in a non-contact mode and the external portion of the RWM was surrounded by air (with a refractive index of 1). The scanning was performed at the rate of 24 kHz and 48 kHz without averaging. Two- and three-dimensional scans of the cochlear microanatomy were acquired through the RWM. The acquisition time was less than 0.1 s for two-dimensional scans and between 2 s and 7 s for three-dimensional scans.

For the purpose of scanning, TB1 and TB2 were held in place on a temporal bone holder (Fig. 4), while TB3 and TB4 were fixated on a plastic container in the optimal position with play-doh modelling clay. The temporal bone holder and the plastic container were placed on the translational stage of the OCT system.

### OCT images storage, analysis and processing

The acquired images were stored as. oct files. They were viewed and analyzed in ThorImage OCT 4.4 software, after adjusting the signal range to [35.7, 60] dB in order to increase the contrast of the image.

Measurements were carried out in ThorImage OCT 4.4 software in TB1 and TB3. Hereby, the refractive index was set to 1.00, for the above-described reasons. In fresh-frozen TB1, we measured the superior-inferior diameter and the thickness of the RWM, the width of the ST and the thickness of the OSL in the cross-section with the largest ST width for these measurements (Fig. 2d). In fixed TB3, we measured the thickness of the RWM as well, in order to compare this value with fresh-frozen TB1.

For further processing, the two-dimensional OCT images and the xz- and yz-cross-sections of the three-dimensional images were exported from the software as. jpeg files. The measurements and the names of anatomical structures have been added to the respective images in the ImageJ software.

### Ethical approval and informed consent

The temporal bones in our study were harvested and used in accordance with the Helsinki declaration from individuals who voluntarily donated their body to research in their testament. The experimental protocols were approved by the Medical Ethics Committee of the University Hospitals of Leuven (approval No. NH019 2016-06-04). The donors were anonymous and no biographical donor data were known. Therefore no additional informed consent was required.

## Supplementary information

Supplementary Information

## Data Availability

All data analyzed during this study are included in this published article and its Supplementary Information files.
