# Supplementary material for: High-resolution Imaging of the Human Cochlea through the Round Window by means of Optical Coherence Tomography
Source: Sci Rep. 2019 Oct 3;9:14271. doi: 10.1038/s41598-019-50727-7 (PMC6776619; doi:10.1038/s41598-019-50727-7)
Supplement: Supplementary file 1 — Supplementary Information [file 41598_2019_50727_MOESM1_ESM.pdf]

Supplementary Information

High-resolution Imaging of the Human Cochlea through the Round Window by means of Optical Coherence Tomography

Anastasiya Starovoyt[1] , Tristan Putzeys[1,3] , Jan Wouters[1] and Nicolas Verhaert[1,2,*]

[1]Research Group Experimental Oto-Rhino-Laryngology, Department of Neurosciences, University of Leuven, Leuven, Belgium. [2]Department of Otorhinolaryngology, Head and Neck Surgery, University Hospitals of Leuven, Leuven, Belgium. [3]Laboratory for Soft Matter and Biophysics, Department of Physics and Astronomy, University of Leuven, Leuven, Belgium. [*]Corresponding author. (email: nicolas.verhaert@kuleuven.be)

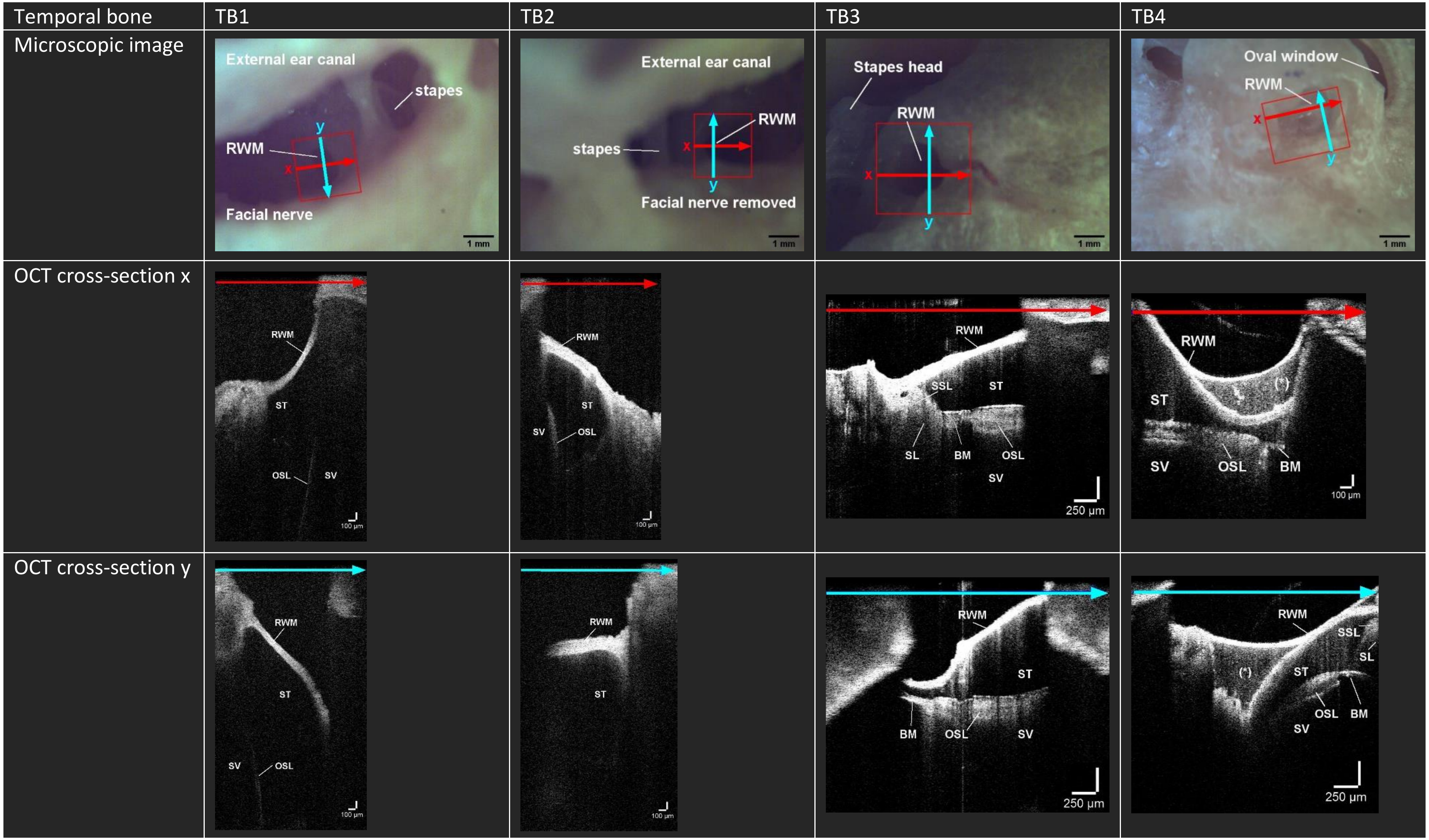
Temporal bone
TB1
TB2
TB3
TB4
Microscopic image
External ear canal
stapes
RWM
Facial nerve
y
x
1 mm
External ear canal
RWM
stapes
Facial nerve removed
Stapes head
Oval window
OCT cross-section x
RWM
ST
OSL
SV
100 µm
SSL
SL
BM
250 µm
(*)
OCT cross-section y

Supplementary Figure 1. Overview of the three-dimensional OCT scans in TB1 to TB4. In all temporal bones, we selected the cross-sectional images through the center of the RWM. The scanned area is indicated with a red rectangle. The red and blue arrows show the position of the cross-section x and the cross-section y respectively (the arrow point always corresponds to the right side of the cross-sectional image). Note the similarity between the corresponding cross-sectional images of TB1 and TB2, TB3 and TB4. The three-dimensional OCT scan of TB3 is different from Figure 3. Note that the cross-sectional images of TB3 and TB4 are almost a mirror image of each other, with exception of the (*) part in TB4. As described in the Methods section, TB4 dried out after fenestrations. In order to improve the resolution of OCT images, the cochlea has been moisturized with tap water prior to imaging. Hereby, we saw that the RWM in TB4 split in two layers, creating the fluid-filled cavity (*) between them. Based on the location of the cavity, the perceived thickness of the RWM layers on the OCT images (Supplementary Figure 1, TB4, cross-section x and cross-section y) and the known structure of the RWM[17], the RWM most likely split at the level of its loose connective tissue. The outer layer of the fluid-filled cavity presumably consists of the outer epithelium of the middle ear with adjacent loose connective tissue, while the inner layer comprises the dense connective tissue with the adjacent inner epithelium[17].
BM: basilar membrane, OSL: osseous spiral lamina, RWM: round window membrane, SL: spiral ligament, SSL: secondary spiral lamina, ST: scala tympani, SV: scala vestibuli.

**Temporal bone**	**TB1**	**TB2**	**TB3**	**TB4**
**Side**	Left	Right	Right	Left
**Aspect**	Fresh-frozen	Fixed	Fixed	Fresh-frozen, dry
**Mastoidectomy**	X	X	X	X
**Posterior tympanotomy**	X	X	X	X
**Removal of the bony overhang around the RWM**	X	X	X	X
**Removal of the facial nerve**	-	X	X	X
**Isolation of the cochlea**	-	-	X	X
**Fenestrations of the cochlea**	-	-	-	X

Supplementary Table 1. Overview of the temporal bones with their side, aspect (fixed of fresh-frozen) and dissection technique. RWM = round window membrane; TB = temporal bone.
